# Understanding oncogenicity of cancer driver genes and mutations in the cancer genomics era

**DOI:** 10.1002/1873-3468.13781

**Published:** 2020-04-28

**Authors:** Eduard Porta‐Pardo, Alfonso Valencia, Adam Godzik

**Affiliations:** ^1^ Barcelona Supercomputing Center (BSC) Barcelona Spain; ^2^ Josep Carreras Leukaemia Research Institute (IJC) Badalona Spain; ^3^ Institucio Catalana de Recerca I Estudis Avançats (ICREA) Barcelona Spain; ^4^ Division of Biomedical Sciences University of California Riverside School of Medicine Riverside CA USA

**Keywords:** cancer drivers, cancer genes, multiscale analysis, personalized medicine, variants of unknown significance

## Abstract

One of the key challenges of cancer biology is to catalogue and understand the somatic genomic alterations leading to cancer. Although alternative definitions and search methods have been developed to identify cancer driver genes and mutations, analyses of thousands of cancer genomes return a remarkably similar catalogue of around 300 genes that are mutated in at least one cancer type. Yet, many features of these genes and their role in cancer remain unclear, first and foremost when a somatic mutation is truly oncogenic. In this review, we first summarize some of the recent efforts in completing the catalogue of cancer driver genes. Then, we give an overview of different aspects that influence the oncogenicity of somatic mutations in the core cancer driver genes, including their interactions with the germline genome, other cancer driver mutations, the immune system, or their potential role in healthy tissues. In the coming years, this research holds promise to illuminate how, when, and why cancer driver genes and mutations are really drivers, and thereby move personalized cancer medicine and targeted therapies forward.

## Graphical abstract

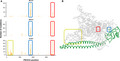

## Abbreviations

**CGC**, Cancer Gene Census

**CRISPR**, clustered regularly interspaced short palindromic repeats

**GTEx**, Genotype–Tissue Expression

**ICGC**, International Cancer Genome Consortiu

**TCGA**, The Cancer Genome Atlas

**TME**, tumor microenvironment

**VUS**, variant of unknown significance

Box 1 **Take‐home messages**

- We are nearing an almost‐complete catalogue of cancer driver genes
- The main drivers were discovered decades ago, but we still do not understand many aspects of their biology
- Cancer driver genes have many variants of unknown significance
- The germline genome interacts with the somatic variants
- Cancer driver genes interact also with each other
- The oncogenicity of cancer driver genes and mutations depends on the tissue and overall context (*e.g.,* germline mutations, immune status of the individual)

The analysis of the first cancer genomes revealed that each tumor had acquired hundreds or even thousands of somatic mutations during its evolution. While at the time there was already a catalogue of genes known to be involved in cancer, whole‐exome and later whole‐genome sequencing of tumor samples provided the opportunity to identify cancer genes in an unbiased and data‐driven way. To that end, dozens of computational biology and bioinformatics groups started developing tools to analyze these large datasets and distinguish the genes that contribute to tumor progression from those that are instead neutral.

Genes in this first category are called driver genes, those in the latter are named passengers and the same nomenclature can be used for both individual mutations and other genetic events. Interestingly, despite the apparent simplicity of this concept, the exact definition of cancer drivers is still debated, as best evidenced by hundreds of papers offering different practical implementation of algorithms identifying them. Most of them are based on the idea that drivers should show evidence of positive selection, which can be defined by a statistically significant difference between an observed number of mutations and those expected by chance. But the background mutation rate and its distribution are not known, and different algorithms use different assumptions to estimate it.

More than a decade and tens of thousands of cancer genomes later, thousands of genes, at some point, have been defined as potential cancer drivers using different algorithms. Nevertheless, there is a list of around 300 genes that is consistently identified in almost all analyses: This core list consists of the most important cancer driver genes and is unlikely to change in the future. Encouragingly, many of these genes were first identified decades ago by molecular biologists and now are being ‘rediscovered’ by unsupervised analyses. So, while we have not yet identified the precise catalogue of cancer driver genes or events, nor do we even agree on their definition, there seems to be a broad consensus about a ‘core’ group.

Besides lacking a ‘final list’ of cancer driver genes, we also do not understand many of the cancer‐relevant features of these genes. Arguably, one of the most important open questions is when a somatic alteration in a cancer driver gene is truly oncogenic, as personalized cancer care often hinges on its answer. Here, we will review the recent efforts that address this question across multiple biological scales. We will first focus on how different mutations within the same cancer driver gene might have different effects. Then, we broaden the scope and summarize recent results supporting the existence of functional interactions between somatic mutations in cancer driver genes and other genetic alterations, either somatic or germline. Finally, we give an overview of the evidence gathered so far about the role of the tissue context in determining the oncogenicity of cancer driver mutations.

## The most common cancer driver genes have been identified

Since the creation of the first Cancer Gene Census (CGC) [1], there have been several major efforts to compile a comprehensive catalogue of cancer driver genes. Most of the recent analyses have exploited data from The Cancer Genome Atlas [2] (TCGA) or the International Cancer Genome Consortium [3] (ICGC) and the integration of several computational tools to identify cancer driver genes [4, 5]. Others, like the aforementioned CGC, have relied on manual curation of the literature [6]. Over the past 15 years, there have been dozens of studies aimed at completing the catalogue of cancer driver genes [7, 8, 9, 10] and, as a result of these efforts, thousands of genes have been suggested to drive cancer growth.

**Fig. 1:**
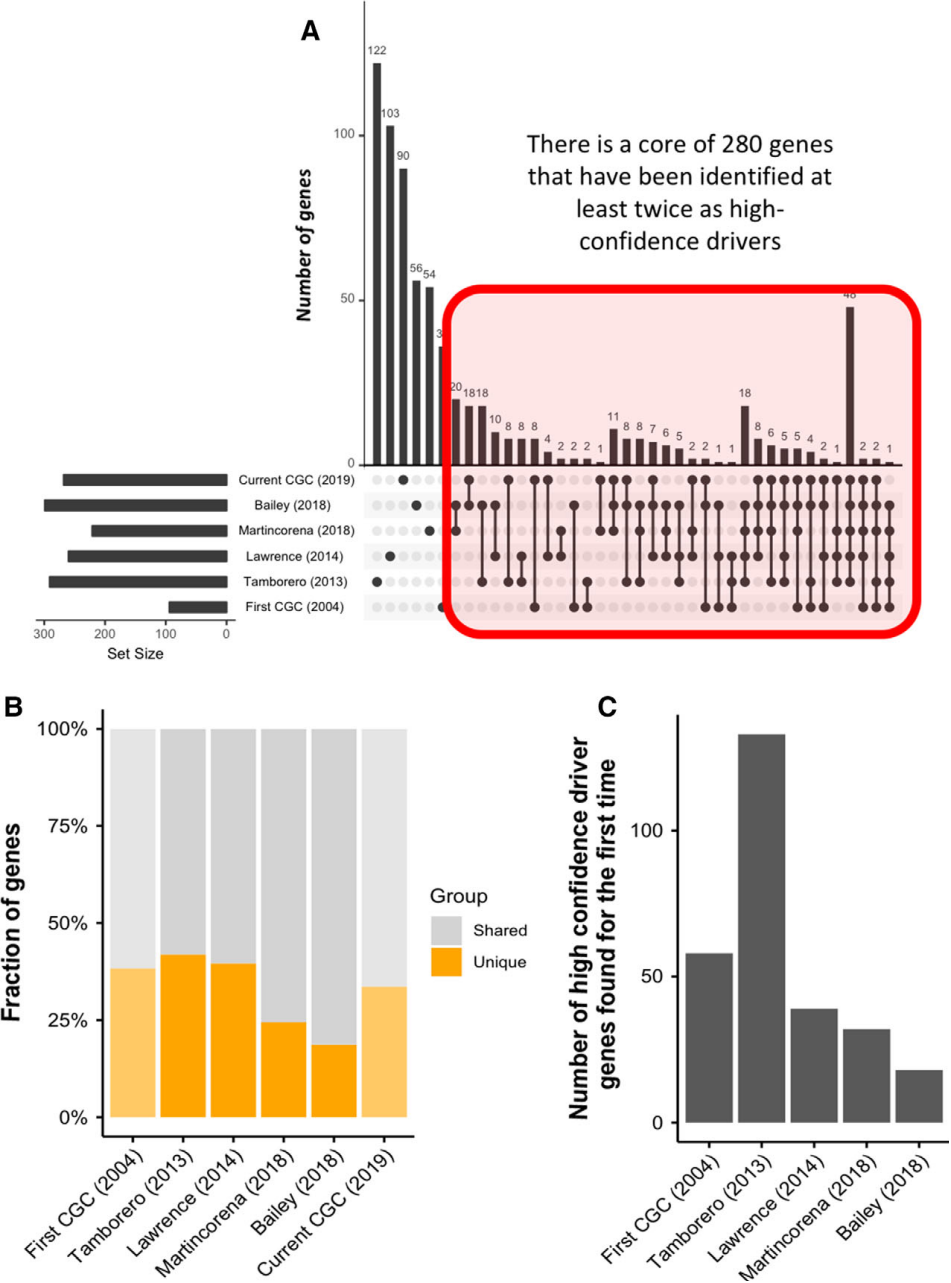
The quest for new cancer driver genes is approaching its end. (A) Upset plot showing the overlap of six different sets of cancer driver genes published during the last 15 years. (B) Barplot showing the fraction of cancer driver genes that is either unique to each set (orange) or found in at least another study (gray). (C) Barplot showing the number of high‐confidence driver genes (i.e., those found at least twice) was found for the first time in the analyzed dataset

To evaluate whether there is a consensus on which genes are true drivers and how much we have learned during the genomic era of cancer, we have compared four of the most cited lists of cancer driver genes that spanned different time points across the last seven years [4, 5, 7, 10], as well as the first [1] (2004) and the current [6] (2019) versions of the CGC (Fig. 1). Of note, the genes linked to cancer only by means of germline mutations or somatic translocations were excluded from both CGC lists, as these are not analyzed by most cancer driver detection tools. These lists together contain 741 genes, and there is a set of 280 genes common to two or more lists. The original CGC contained 94 genes (after the filtering mentioned above). Of these, 26 have been consistently found in all subsequent studies, including ‘classical’ cancer driver genes such as TP53, KRAS, NRAS, HRAS, EGFR, and BRAF. The remaining 68 are divided between those found at least once in the following 15 years (32 genes), and those that were never re‐identified as somatic drivers (36). Although one might think that this last group of genes represents false positives, it includes genes with known germline roles in cancer such as FANCCA, FANCD2, FANCF, XPC, ERCC3, and ERCC5.

There are 48 genes that were not part of the original CGC but have been found in all following studies and are now included in the CGC. Among them are some of the most important discoveries from the first cancer genomics era, for instance, B2M, STAG2, IDH1, IDH2, ARID1A, SPOP, KDM6A, RHOA, CASP8, or PIK3R1, as well as genes that were initially linked to cancer only via translocations and are now known to be altered by somatic single nucleotide variants, such as EP300.

Notably, the number of unique genes found in each list has been shrinking over the years, from 122 unique genes (Tamborero *et al.* [5], 2013) to 54 and 56 genes (Martincorena *et al.* [7] and Bailey *et al.* [4], 2018), suggesting that the number of false positives is decreasing over time and that the identification of new cancer driver genes is plateauing (Fig. 1B). In fact, most of the cancer driver genes found in two studies were discovered in the first TCGA analyses (Tamborero *et al.* [5] and Lawrence *et al.* [10]; Fig. 1C). Thus, it seems likely that the most common cancer driver genes have already been discovered. However, as we will see in the following sections, this does not mean that we understand their role in oncogenesis.

## Variants of unknown significance in cancer driver genes

The type and distribution of somatic mutations within cancer driver genes strongly depends on their oncogenic role [11]. Oncogenes usually have clear hotspots that are strongly enriched in somatic activating missense mutations (*e.g.,* KRAS G12, PIK3CA E545, BRAF V600). On the other hand, tumor suppressor genes tend to be affected by frameshift or truncating mutations that completely abrogate the function of the encoded protein. Tumor suppressor genes can also have somatic mutation hotspots that inactivate their function, but these are rarer and tend to affect genes that can be both oncogenes and tumor suppressors, depending on the context. Hence, it is easy to know whether a mutation is oncogenic, as identifying frameshift and truncating mutations is relatively straightforward and there are catalogues of which missense mutations in a given hotspot have oncogenic effects [12].

**Fig. 2:**
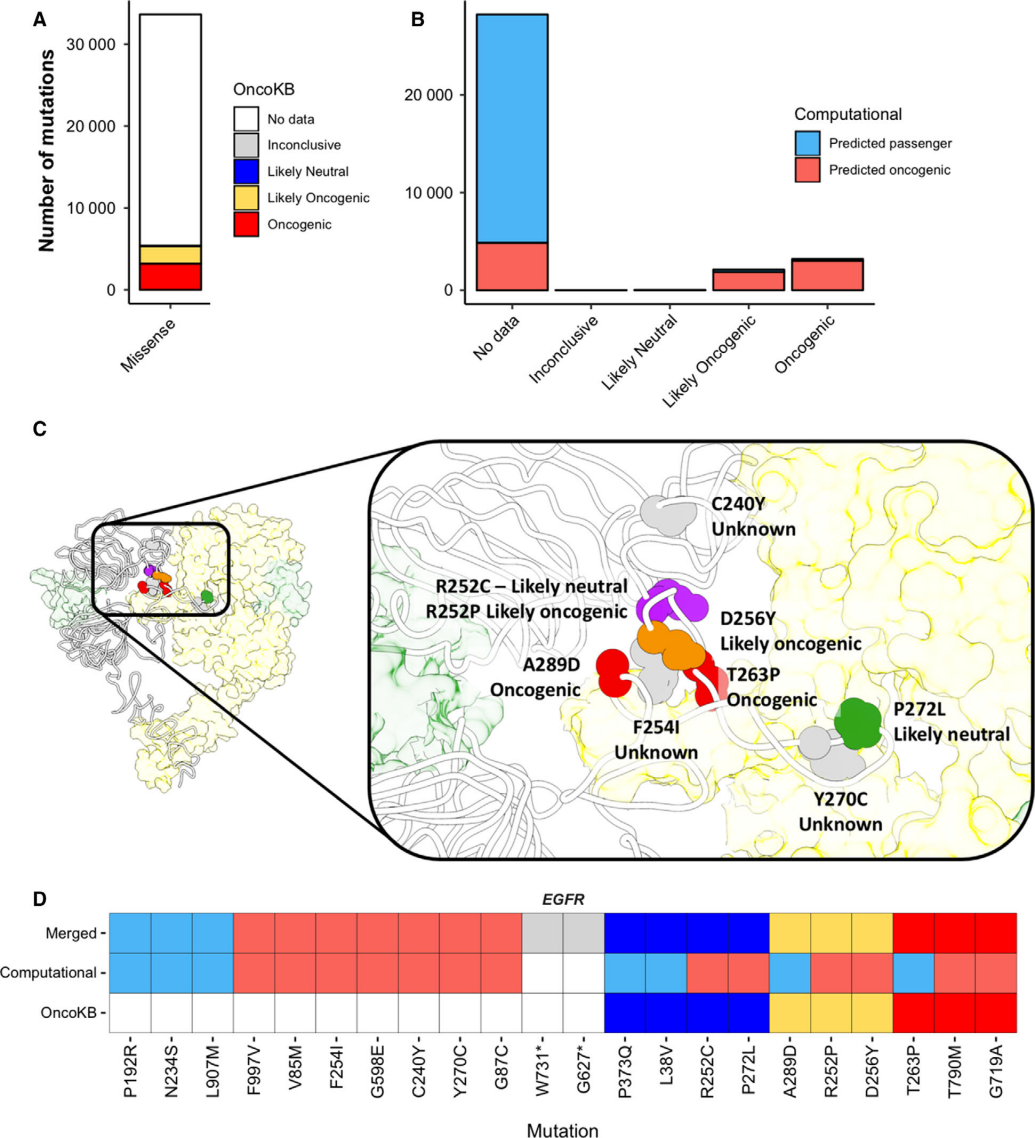
Predicting the oncogenicity of somatic mutations. (A) Number of missense somatic mutations in cancer driver genes in TCGA, according to their oncogenicity annotation in OncoKB. (B) Computational prediction of the oncogenicity of all somatic missense mutations in cancer driver genes found in TCGA. Each column represents an OncoKB category. (C) Subset of somatic missense mutations in the dimerization interface of EGFR found in glioblastoma and lower grade glioma patients from The Cancer Genome Atlas. Mutations are colored according to their OncoKB annotations. (D) A consensus classification of some somatic mutations in EGFR, including all those from panel a. Each tile is colored according to the classification of the corresponding mutation as annotated in OncoKB (bottom), a computational analysis (middle) and a potential consensus between the two (top)

Nevertheless, there are many cases where a tumor carries a variant of unknown significance (VUS) in a cancer driver gene. These are often missense mutations located in tumor suppressor genes or outside the known mutational hotspots in oncogenes. To put this in perspective, patients from TCGA have a total of 44 607 somatic mutations in cancer driver genes. Only 5435 of these are in OncoKB [12], leaving the oncogenicity of the remaining 39 172 (88%) unknown. Even if we assumed that all frameshift and truncating mutations in cancer driver genes are oncogenic, there would remain 28 238 missense mutations of unknown significance (63% of all somatic mutations in these genes; Fig. 2A).

There are two main approaches to analyze the role of these variants of unknown significance: experimental and computational. Experimental methods are more time consuming, but recent advances in saturation mutagenesis, CRISPR technology and automation of cell culture make the high‐throughput analysis of thousands of mutations more accessible to researchers. In fact, a subset of cancer driver genes has been analyzed using deep mutational scans that test virtually all potential mutations in a certain gene. This has been done, for example, for TP53 [13, 14], BRCA1 [15], HRAS [16], PTEN [17], and MAPK1 [18]. There are also other analyses that, while not comprehensively studying individual proteins, have reported the oncogenicity of thousands of somatic mutations in dozens of different genes [19, 20].

Computational methods have also been extensively explored. Their main advantages are that they are orders of magnitude faster and less expensive than experimental methods, allowing researchers to study virtually any mutation. For example, using 12 different computational tools, we predicted the role of all missense mutations in the cancer driver genes from TCGA [4]. These predictions had a large agreement with OncoKB [12] annotations (Fig. 2B), with the advantage that they gave information on 28 238 missense somatic mutations not annotated in OncoKB. Importantly, 4864 missense mutations in cancer driver genes from TCGA with no data in OncoKB are predicted to be oncogenic (Fig. 2B).

According to the type of data employed, there are four different groups of computational methods to predict the effects of VUS (Table 1). Group I consists of methods that use sequence information to distinguish between benign and disease‐associated mutations. These tools have not been designed specifically for cancer but, instead, to separate mutations associated with rare diseases, diabetes, asthma, and cancer, among others, from those that are benign. Methods in Group II also use sequence information but have been trained specifically to distinguish between passenger and driver mutations using cancer‐specific data. The distinction between disease‐associated (Group I) and oncogenic mutations (Group II) seems important, as the performance of each group of methods in separating passenger and driver mutations is different [4]. Group III includes those methods that predict cancer driver mutations using data from three‐dimensional protein structures. These methods seem to be more accurate than those that use only sequence data [4], but they can only be applied to mutations where the structure is experimentally determined or can be reasonably modeled. Finally, there is a fourth group of methods (Group IV) that combine linear and three‐dimensional features using machine‐learning approaches.

**Table 1:** List of driver prediction algorithms and their classification (see text for details)

Method	Group	References
SIFT	I	[79]
PolyPhen‐2	I	[80]
MutAssessor	I	[81]
transFIC	I (Ensembl)	[82]
CADD	I (Ensembl)	[83]
MCAP	I	[84]
REVEL	I (Ensembl)	[85]
VEST	I	[86]
FATHMM	II	[87]
CanDrA	II	[88]
CHASM	II	[89]
ParsSNP	II	[90]
HotMAPS	III	[91]
HotSpot3D	III	[92]
3DHotspots.org	III	[93]
e‐Driver3D	III	[94]
CATH‐FunFams	III	[95]
CHASMplus	IV	[96]

Whenever possible, it is important to couple computational predictions with experimental data. For example, most EGFR mutations in brain tumors (glioblastoma and lower grade glioma) are located near its dimerization interface (Fig. 2C). However, we only have experimental annotations for a small subset of all of these mutations. Putting side by side the experimental results and the computational predictions (Fig. 2D), a reasonable agreement between the two, albeit with some discrepancies, comes to light.

## Historical contingency and cancer driver genes

The paths that life can follow are constrained by previous events, including seemingly inconsequential genetic variations. This phenomenon, also known as historical contingency [21], has implications in tumor evolution, as a mutation might be beneficial in a certain genetic background and detrimental in another. Similarly, a tumor might only be able to access certain genotypes, if it has previously acquired other mutations. As we will see in the following paragraphs, cancer cells are also subject to historical contingency: The evolutionary paths that a tumor can explore depend on the genetic variations it has acquired over time [22].

The genetic background of a cancer cell includes both the somatic variants it has acquired over time and the germline variants that, by definition, were present before any somatic variant ever occurred. For example, each individual carries between 20 000 and 30 000 coding germline variants, some of which even completely disrupt entire proteins [23]. Moreover, each individual also has hundreds or thousands of germline noncoding variants that influence gene expression, including cancer drivers [24]. Finally, once somatic evolution begins, it can add hundreds of coding somatic variants and thousands of noncoding ones, and the order in which some of them are acquired will determine the final phenotype of the cancer cell.

The germline genome can interact with cancer driver mutations both *in cis* and *in trans* (see [25] for an in‐depth review of the topic). Cis interactions are those that happen between variants of the same locus, and such functional interactions have been described for a few cancer driver genes. One of the first examples of germline–somatic cis interactions was described for the *JAK2* somatic mutation V617F. This mutation, which transforms JAK2 into driver of myeloproliferative neoplasms, is much more likely to happen in the haplotype with the minor allele of rs12343867 [26]. Similar interactions have been described for somatic *EGFR* exon 19 deletion, which is three times more likely in individuals with the minor rs712829 allele, located in the gene promoter [27]. Finally, it is worth noting that deep mutational scans could help discover many cis interactions between germline and somatic variants. This has been recently shown in *TP53*, where the effect of dozens of somatic missense mutations depends on the allele of the germline ultra‐rare rs35163653 (MAF < 1e‐5, p.V217M) [14].

Cancer driver mutations can also interact in trans with germline variants. This phenomenon has been recently explored using data from TCGA [28], identifying 28 germline variants associated with changes in the frequency of 20 somatic variants, suggesting an interaction between the two. One of the better characterized interactions in that study is that between the germline variant rs25673 and somatic *PTEN* mutations. Individuals with the minor germline allele at rs25673 are five times more likely to have a *PTEN* somatic mutation in their tumors. The likely reason is that these individuals have an intrinsic higher expression level of STK11 and/or GNA11. When adding information at the pathway or protein interaction network, the possible connection between these two genes becomes apparent, as they are both upstream of PTEN, so their higher expression could make a somatic PTEN mutation more oncogenic than it would be in a different genetic background [28]. These results highlight the importance of accounting for protein interactions and signaling pathways, already routinely used by many approaches that analyze either germline [29, 30, 31, 32, 33] or somatic [34, 35, 36, 37, 38, 39] variants alone, when integrating both.

Interactions between the germline and somatic genomes could also have consequences for genetic risk prediction. For example, 25 germline SNPs associated with glioma and glioblastoma have been recently tested for their association with the most frequent somatic alterations in these cancer types: IDH1 R132H and 1p/19q deletions [40]. Based on this analysis, the authors were capable of building a polygenic risk score that predicted not only risk to glioma but, specifically, to IDH1‐driven glioma. Given the significant biological differences between IDH1‐mutated and IDH1 wild‐type brain tumors, whether this can be extended to other combinations of cancer types and somatic driver events remains to be seen. Nevertheless, these are significant first steps toward a comprehensive understanding of the interactions between the germline genome of cancer patients and the somatic mutations acquired by their tumors.

Sex of the patient and their ancestry also correlate with the type and outcomes of many cancers, highlighting the importance of historical contingency and germline–somatic interactions in tumor evolution. The prevalence of many cancer types differs between males and females: Thyroid cancer is three times more likely to occur in women than in men, whereas bladder cancer is twice more likely in men than in women, for example. While this could be attributed to differences in the environment of each gender, such as prevalence of smoking or differences in hormone levels, multiple lines of evidence also point to genetics [41]. For example, the frequency of certain somatic driver mutations depends on the sex [42]. Also, the sex chromosome X contains multiple oncogenes and tumor suppressors that can contribute to sex bias and other cancer phenotypes by escaping X‐inactivation in females [43, 44]. Similarly, the genetic ancestry of an individual also correlates with the prevalence of cancer driver mutations. For example, somatic mutations in TP53 and CCNE1 are more common in cancer from African Americans than in those from Europeans, whereas the opposite is true for somatic variants in PI3KCA [45].

Finally, the order in which somatic mutations occurred can also influence their phenotype. One of the first examples of this phenomenon was described in a model of colorectal cancer, where tumors only develop when somatic mutations are acquired in a precise order [46]. Similarly, renal tumors seem to be constrained to only few evolutionary pathways [47]. Which one of these pathways is taken by the tumor seems to be determined by the initial somatic driver event. Recently, using TCGA data, this has been systematically studied in dozens of different cancer types. The TCGA analysis provided indirect evidence of somatic historical contingency, as somatic mutations can either be clonal (i.e., they are acquired in the primary neoplasm and are thus present in all tumor cells) or subclonal (i.e., they are acquired after the tumor started its expansion and are only present in a subset of cells) [22]. An even more dramatic example is seen in myeloproliferative neoplasms. There are two key driver genes that, when mutated somatically in a myeloid progenitor, they can potentially become malignant: JAK2 and TET2. However, the final phenotype of the patient depends on the order in which these mutations are acquired. If a mutation in JAK2 is acquired before the TET2 mutation, there is an expansion of hematopoietic stem and progenitor cells as well as a blockage of the expansion of erythroid progenitors. On the other hand, if the order is inverted, there is an expansion of megakaryocytes and blockage of the hematopoietic cell pool [48].

Overall, it seems clear that the evolutionary trajectories of cancer cells are constrained by the genetic variants already present in their genomes, regardless of their somatic or germline origin. Understanding and predicting such constraints could have significant impact in both, the diagnosis (as seen for the polygenic risk scores for IDH1 mutations) as well as the treatment of cancer [49].

## The relationship between the immune system and cancer driver genes

Following the explosion of immune‐based therapies to treat cancer, we are now also improving our understanding of the complex relationship between the immune system and somatic cancer driver mutations. The relationship between the two seems to be bidirectional, as the immune system has a strong effect in determining which cancer driver mutations can happen in a cancer patient [50] while, at the same time, the presence of certain driver mutations correlates with the quantity and composition of immune cells in the tumor microenvironment (TME) [51].

Regarding the influence of the immune system in the presence of cancer driver mutations, it is mostly mediated by the fact that all somatic mutations can create neoantigens: peptides that have not been previously presented to immune cells via HLA and that, therefore, can be identified as foreign by the immune system. If presented in the appropriate context, these neoantigens can trigger an immune response that ends in the elimination of the cell that carries them, a process known as immunoediting. As any other somatic mutation, those located in cancer driver genes are not exempt from immunoediting. In fact, driver somatic seem to have been selected to be poorly presented in the majority of both, class I [50] and class II HLA alleles [52]. At the individual patient level, a common immune‐evading mechanism of cancer cells is the loss of expression of HLA alleles that can present their driver mutations [53]. In fact, the effect of immunoediting is so strong that it can be seen at the population level: The frequency of a cancer driver mutation is negatively correlated with the frequency of the HLA alleles that present the peptides derived from it [52].

However, as explained above, the presence of certain cancer driver mutations correlates with differences in the quantity and composition of the immune infiltrate in the tumor microenvironment [51, 54]. Whether these correlations are causal or not remains to be seen in most cases, but some molecular mechanisms have been proposed for a few cases. For example, somatic mutations in driver genes with known roles in immune signaling, such as CASP8 or HLA, are generally associated with higher levels of immune cells in the TME, likely because these mutations are, indeed, an immune‐evading mechanism. In other cases, however, the connection can be more obscure, as in the case of colorectal tumors with KRAS mutations. These tumors are known to have low levels of immune infiltrate and be resistant to immune‐checkpoint blockade. These phenotypes could be due to KRAS repressing the interferon regulatory factor 2 (IRF2), leading to high CXCL3 expression and the recruitment of myeloid‐derived suppressor cells to the tumor microenvironment [55]. Another group of cancer driver mutations with a likely mechanism to link them with changes to the immune infiltrate of the TME are those in the Wnt/beta‐catenin pathway. Tumors with mutations in this pathway, particularly in CTNNB1, have low levels of immune cells across multiple cancer types, likely through the exclusion of BATF3‐derived dendritic cells from the TME [56]. Overall, however, the relationship between somatic driver mutations and the immune response against cancer cells will likely be an important topic in the coming years.

## Interactions between the tissue of origin of the tumor and cancer driver genes

**Fig. 3:**
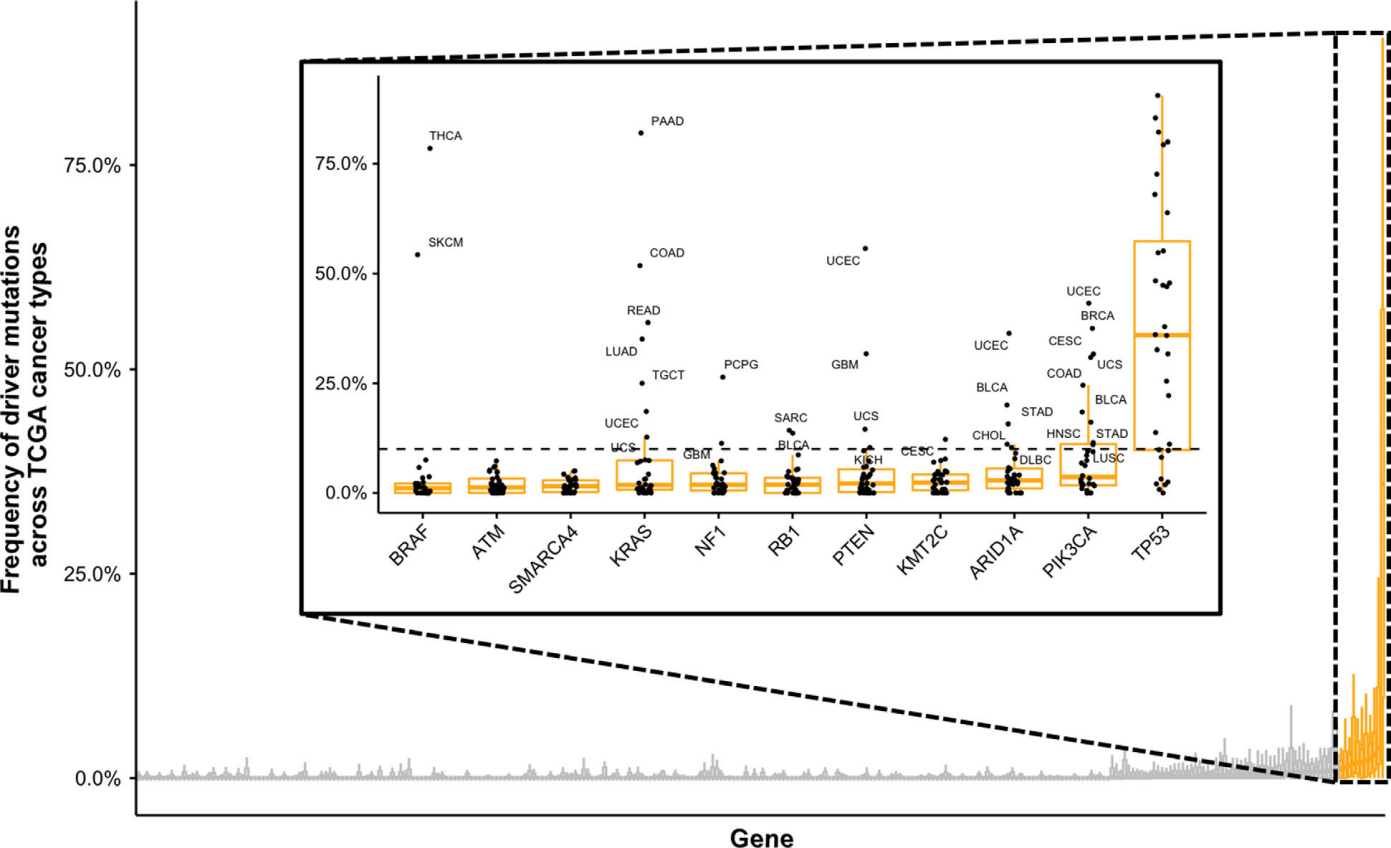
Cancer driver genes are tissue‐specific. Each boxplot in the x‐axis represents the distribution of mutation frequencies for a cancer driver gene across the 33 cancer types of TCGA. Out of the ten most frequently mutated cancer driver genes (average across all tissues) are highlighted in orange. Only *TP53* has an average mutation frequency above 10%

The cell of origin of the tumor also influences the oncogenic potential of cancer driver mutations. This is evident, for example, in the differences in the prevalence of a given mutation across different cancer types (Fig. 3). Out of the 299 cancer driver genes recently described in the Pan‐Cancer Atlas analysis of TCGA, only *TP53* has a median somatic mutation frequency over 10% across all cancer types (35%) and only ten other genes have a median frequency above 1% (ARID1A, ATM, BRAF, KMT2C, KRAS, NF1, PIK3CA, PTEN, RB1, and SMARCA4). The remaining 288 cancer driver genes have a median mutation frequency below 1%. Moreover, the mutation frequency of each cancer driver gene is highly variable. For example, BRAF has a frequency above 50% in melanoma and thyroid adenocarcinoma but below 10% in all other cancer types (Fig. 3). Something similar happens with EGFR, with relatively high mutation frequencies in glioblastoma (24%), lung adenocarcinoma (7%) and glioma (6%), but below 1% in the remaining 30 cancer types. Overall, there are 43 cancer driver genes that have a mutation frequency above 10% in at least one cancer type, but whose median frequency is below 1%.

Moreover, even if somatic driver mutations are shared across cancer types, their role and interactions can differ depending on the tissue. This is the case of BRAF V600E, which is present in melanoma and colorectal adenocarcinoma patients. Yet, these two tumor types differ in their sensitivity to the BRAF inhibitor vemurafenib. Melanoma patients initially respond very well to the treatment [57], but colorectal cancer patients do not [58]. Similarly, some driver mutations seem to cooperate in some cancer types but are mutually exclusive in others. This is the case, for example, of KRAS and TP53, which co‐occur in pancreatic adenocarcinoma but are mutually exclusive in lung adenocarcinoma [59].

**Fig. 4:**
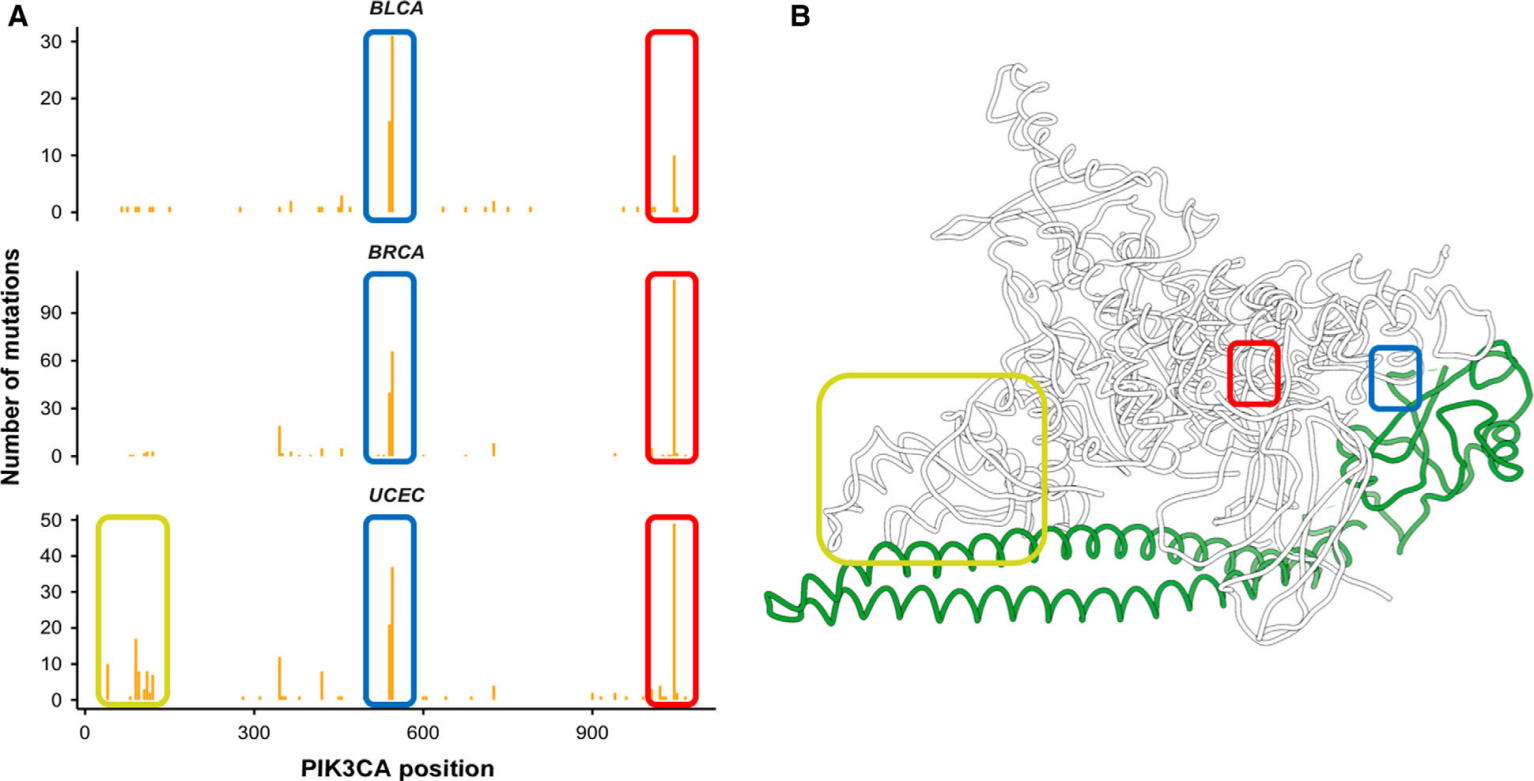
Mutation‐hotspot prevalence of PIK3CA depends on cancer type. (A) The mutation frequency of different hotspots (E545, in blue, H1047, in red, and the N‐terminal domain, in yellow) differs depending on the cancer type (left). (B) Location of the different hotspots in the PIK3CA–PIK3R1 dimer (in white and green, respectively) structure from PDB file 3HMM

Cancer driver genes can also show different mutational patterns depending on the cancer type [60]. These differences could be caused by the distinct mutational processes active in each cancer type. This has been shown in TP53, where the prevalence of the different missense mutations in different cancer types depends, not only on the effect of the mutation, but also on the mutational signature active in that cancer type [13]. Another possibility is that the molecular processes altered by different mutations within the same gene can have varying tissue‐specific degrees of oncogenicity, as could be the case for PIK3CA mutations [61, 62] (Fig. 4).

All of the above is likely to have a significant impact also on personalized cancer care. For example, germline mutations in BRCA1 and BRCA2 predispose to multiple cancer types, specifically to ovarian and breast cancer in women and prostate cancer in men. Using a synthetic lethality screen, Jonsson et al. discovered that breast cancer cells with mutations in these two genes are sensitive to PARP inhibitors [63]. Since BRCA mutations are relatively common in many other cancer types, it was hoped that the synthetic lethality interaction between PARP and BRCA would also extend to these other cancer types. Nevertheless, it seems that the lethal interaction only happens in specific cell lineages, specifically the same ones where germline BRCA1 and BRCA2 mutations predispose to cancer. This highlights the importance of tissue specificity, not only to understand oncogenesis [64, 65], but also in determining the success of targeted therapies [66].

## Healthy cells can carry driver mutations

One of the most paradoxical and surprising results about cancer driver genes is the discovery of healthy cells with somatic driver mutations. This was first shown in skin cells carrying the BRAF V600E mutation [67], but has been later extended to cells from the esophagus with NOTCH1 truncating mutations [68], with more recent studies extending the work to healthy colon [69], the colon of patients with inflammatory bowel disease [70], or the endometrium [71]. In fact, two analyses have studied somatic mutations in the entire human body [72, 73]. The authors identified somatic mutations from RNAseq coming from 29 different tissues of over 500 healthy donors that were part of the GTEx project (https://www.gtexportal.org/home/). Virtually all tissues seemed to carry cancer driver somatic mutations in some individuals, even if none of them had been diagnosed with cancer. The most extreme example of this phenomenon is probably the recently described role of somatic PTEN, KMT2D, and ARID1A mutations in healthy liver [74]. These genes are known cancer drivers, but recently Zhu et al. showed that, under certain circumstances, somatic mutations in these genes are actually beneficial to the homeostasis of the liver [74]. Liver cells that have somatic mutations in PTEN, KMT2D or ARID1A have higher fitness. When the liver is under stress and needs to be regenerated, these cells can expand faster than their nonmutated counterparts and, thus, regenerate the tissue in less time.

Overall, it seems that somatic cancer driver mutations are pervasive in healthy organs. But, in that case, how is it possible that all of us have thousands of cells with oncogenic mutations and not develop cancer? The most accepted theory to explain this is that a cell requires multiple somatic insults before becoming malignant. This agrees with observations from premalignant stages of certain tumors, where cells already have some driver mutations, but it is not until they reach a minimum threshold, or certain specific driver mutations that they actually become malignant [75]. This is the case, for example, of age‐related clonal hematopoiesis, which is a natural phenomenon in which the pool of hematopoietic stem cells becomes dominated by a few clones as individuals age. When such clonal expansion is accompanied by somatic mutations in driver genes, it can eventually cause acute myeloid leukemia (AML). However, not all driver mutations carry the same risk to cause AML: While TP53 and U2AF1 significantly increase the risk of AML, mutations in DNMT3A or TET2 seem to lead to less aggressive cell phenotypes [76]. Moreover, having two or more of these mutations increases the risk proportionately [76]. Along the same line, most tumors from adult patients harbor between 5 and 10 cancer driver mutations irrespectively of their overall mutation rate [77], suggesting that many tumors need a minimum number of driver mutations before becoming oncogenic. However, another interesting alternative is that the germline genome could modulate the oncogenic potential of somatic mutations. As we have shown before, there is evidence of interactions between somatic and germline variants, so it is possible that driver mutations are only oncogenic when they happen in the right germline genetic background. Finally, as is oftentimes the case, all of these mechanisms are not mutually exclusive but, in fact, are likely interacting with each other.

## Conclusions and Perspectives

As we near the end of the beginning of cancer genomics, new questions emerge around the role of cancer driver genes and their associated somatic mutations. One of the most pressing questions that we need to answer is, probably, which mutations are truly oncogenic and which are not, as many aspects of personalized cancer care hang from it.

Here, we have reviewed the features that seem to influence the oncogenic role of cancer driver mutations. First, we have shown that cancer driver genes have many variants of unknown significance, many of them potentially benign from the clinical point of view. However, although new experimental methods, such as deep mutational scans, can give us insights into the oncogenic potential of virtually all mutations in a cancer driver gene, computational tools are still the only practical alternative in most cases.

Then, we have reviewed the recent evidence about the role of historical contingency and interactions with the germline genome in determining the oncogenicity of cancer driver mutations. The same somatic mutation in a cancer driver gene might have different effects depending on which other genetic variants are already present in the cell. This includes both inherited germline variants, as well as other somatic variants that the (pre)cancerous cell has acquired over time. Moreover, the genetic background of the patient, namely the pre‐existing germline variants, is also likely to affect the oncogenicity of the somatic mutations that happen later in life [78], as evidenced by the differences in somatic mutation patterns in individuals with different sex or ancestry. While we already have numerous examples of such phenomena, we are only beginning to grasp its importance.

We have also discussed the importance of the tissue where driver mutations arise. All cancer driver genes, with the exception of the omnipresent TP53, are frequently mutated only in a single or few tissues. Moreover, as shown for PIK3CA and EGFR, the mutation patterns within a gene can also change depending on the cancer type. This, together with evidence that the same driver mutation in different tissues might lead to very different phenotypes (such as drug sensitivity as in the case of BRCA1 and BRCA2), highlights the tissue of origin of somatic mutations must be taken into account in order to properly assess their oncogenic roles.

Another important question that we will need to address in the coming years is the bidirectional relationship between the immune system and cancer driver mutations. Understanding this relationship can be key to find, among others, new drug combinations that extend the scope of immune‐based therapies.

Finally, we have also discussed the growing evidence showing that somatic mutations, including those linked to cancer, seem to be pervasive throughout the body of healthy individuals. This can potentially be explained if each cancer cell would require a minimum amount of driver mutations to become tumorigenic. However, the sheer number of cells that seem to carry potentially oncogenic mutations, together with the surprising results showing the regenerative role of PTEN, ARID1A, and KMT2D somatic mutations in healthy liver, suggest that other phenomena are likely intervening in the process.

In conclusion, while we have probably already identified the core cancer driver genes, in the coming years addressing all of these questions will help understand how, when, and why cancer driver genes and mutations are really drivers.
